# The association between Ki-67 expression and survival in breast cancer subtypes: a cross-sectional study of Ki-67 cut-point in northern Thailand

**DOI:** 10.1186/s12885-025-13724-w

**Published:** 2025-02-25

**Authors:** Phanchaporn Wongmaneerung, Imjai Chitapanarux, Patrinee Traisathit, Sukon Prasitwattanaseree, Wisanu Rottuntikarn, Areewan Somwangprasert, Chagkrit Ditsatham, Kirati Watcharachan, Pitchayaponne Klunklin, Wimrak Onchan

**Affiliations:** 1 Division of Head Neck Breast, Department of Surgery, Chiang Mai University, Chiang Mai, Thailand; 2 Clinical Surgical Research Center, Chiang Mai University, Chiang Mai, Thailand; 3 Northern Thai Research Group of Radiation Oncology (NTRG-RO), Faculty of medicine, Chiang Mai University, Chiang Mai, Thailand; 4 Chiang Mai Cancer Registry, Faculty of Medicine, Chiang Mai University, Chiang Mai, Thailand; 5 Division of Radiation Oncology, Department of Radiology, Chiang Mai University, Chiang Mai, Thailand; 6 Data Science Research Center, Department of Statistics, Faculty of Science, Chiang Mai University, Chiang Mai, Thailand; 7 Research Center in Bioresources for Agriculture, Industry and Medicine, Chiang Mai University, Chiang Mai, Thailand; 8 Department of Pathology, Chiang Mai University, Chiang Mai, Thailand

**Keywords:** Breast cancer, Ki-67, Cut-off, Subtype

## Abstract

**Background:**

Breast cancer is a major health concern worldwide, and Ki-67 level index is a prognostic factor that indicates tumor proliferation and predicts survival outcomes. However, the standard Ki 67 cut-off level varies between local laboratories, and in Thailand, there is no established optimal cut-off level.

**Objective:**

This study aimed to determine the optimal cut-off point for Ki-67 expression and investigate the association between Ki-67 levels and other prognostic factors with 8-year overall survival.

**Method:**

A retrospective review of Ki-67 levels was conducted in non-metastatic breast cancer patients treated at Maharaj Nakorn Chiangmai hospital from January 2013-December 2015, including 507 breast cancer patients.

**Results:**

The ROC curve analysis identified the optimal Ki-67 cut-point as *≥* 30%, with 75% sensitivity and 48.85% specificity. Age over 60 was associated with higher mortality regardless of cancer stage. Locally advanced staging, nodal involvement, Ki-67 *≥* 30%, and triple-negative subtype correlated with poorer survival. Even after adjustments, these factors remained significant in prognostic evaluation. Chemotherapy notably improved survival, especially in high Ki-67 (*≥* 30) patients. However, this effect was not seen in low Ki-67 patients. High Ki-67 patients receiving chemotherapy showed improved survival in early-stage, node-negative cases compared to those who did not receive chemotherapy. HER2-positive patients with high Ki-67 benefited from chemotherapy, but statistical significance was not reached in hormone-positive patients.

**Conclusion:**

This study identified the optimal cut point for Ki-67 in Northern Thailand as 30%. Patients with KI-67 above 30% show significantly lower 8-year survival rates. This is especially relevant for low-risk patients, like those with hormonal subtypes or early-stage nodal negativity. In these cases, KI-67 becomes crucial for treatment decisions. Our study not only aids Northern Thailand’s understanding but also aligns with broader research, emphasizing KI-67’s vital role in planning treatment for low-risk breast cancer patients.

## Introduction

Breast cancer is the most prevalent cancer among females globally, with approximately 2.26 million cases diagnosed in 2020, accounting for 24.5% of all cancer cases, and the leading cause of cancer-related deaths in females, accounting for 15.5% [1]. Several prognostic factors, such as tumor size, axillary lymph node (AXL) metastasis, hormonal status, Her-2 expression, and Ki-67 expression, determine the appropriate treatment for each breast cancer case. Breast cancer is categorized into subtypes based on molecular testing using IHC, and these subtypes are crucial in predicting the disease’s prognosis and suggesting the appropriate treatment decision [2]. Ki-67 is a marker that indicates tumor proliferation and is considered a crucial prognostic factor. Its expression is used to subdivide breast cancer into luminal A and Luminal B subtypes and plays a crucial role in predicting the recurrence rate and survival outcomes in early-stage breast cancer [2–5]. Differences in Ki-67 detection and interpretation methods across laboratories have caused variations in detection results, reducing the accuracy of breast cancer subtyping. According to the 2011 St. Gallen consensus [6] Ki-67 level index is significant in distinguishing between luminal A and Luminal B subtypes, with the recommended cut-off point being 14% by the ROC method and a gene expression profile– defined gold standard [7]. However, some studies suggest that the optimal cut-off point for Ki-67 expression is 20% [8–10] while others indicate that a cut-off of 30% provides a better prognostic value [11]. The St. Gallen consensus of 2013 and 2015 stated that the interpretation of Ki-67 levels should be based on the standards set by the local laboratory [12, 13]. The use of Ki-67 immunohistochemistry (IHC) to assess proliferative activity remains a topic of debate. While Ki-67 scores are widely recognized for their strong prognostic value and their ability to predict the benefit of adding cytotoxic chemotherapy in cases with high scores, establishing a universally applicable cut-off point has been challenging. This difficulty arises due to the continuous nature of Ki-67 expression and various preanalytical and analytical obstacles that hinder standardized evaluation. The primary objective of this study is to determine the optimal cut-off point for Ki-67 for prognostic value in breast cancer patients in Northern Thailand. The secondary aim is to examine the association between Ki-67 levels and other prognostic factors with 8-year survival outcomes for breast cancer.

## Materials and methods

A total of 507 non-metastatic breast cancer patients who underwent treatment between January 2013 and December 2015 at Maharaj Nakorn Chiang Mai Hospital were included in this study after obtaining ethical approval (SUR-2565-08862 Research ID:8862).Inform consent was waived by the ethic committee due to retrospective study but compliance with the Declaration of Helsinki. The study recorded general characteristics such as sex, age, staging, tumor histologic grading, hormonal status, HER-2 status, Ki-67 level index (Ki-67 LI), and treatment received (such as chemotherapy, anti-HER2) from the Chiangmai cancer registry and reviewed the patients’ medical records. The survival status of patients as of July 1, 2021, was determined, and patients without available death information were assumed to be alive.

### Immunohistochemistry study

Representative formalin-fixed, paraffin-embedded tissue sections of the tumors were cut in slices 3-µm-thick and processed following standard procedures for estrogen receptor (ER), progesterone receptor (PR), HER-2, and Ki-67 immunohistochemistry staining. Heat-induced antigen retrieval was performed with Tris-EDTA buffer (pH = 9), and the slides were stained with monoclonal antibodies against ER and PR by a labeled streptavidin-biotin (LSAB) system (ER Clone SP1 and PR Clone 1E2; Roche). HER-2 staining was done with a monoclonal antibody against HER2 4B5 oncoprotein (Roche), and Ki-67 staining was performed with the MIB-1 clone (1:500; DAKO, ). IHC staining was carried out in a BenchMark ultra autostainer (Ventana Medical System, Tuscon, AZ) using an Ultraview detection kit. All the immunostained slides were reviewed and evaluated in accordance with the current American Society of Clinical Oncology (ASCO)/College of American Pathologists (CAP) guidelines. Among patients with HER-2 2+, the DISH method was performed to determine whether the patient was HER-2 positive or negative. The molecular subtype was categorized by IHC.

The patients were categorized into 4 groups: (1) hormonal receptor positive and HER-2 negative were defined as a hormone-positive group, (2) hormonal receptor negative and HER-2 positive was defined as an HER-2 enriched group, (3) hormonal receptor negative and HER-2 negative were defined as a triple negative group (TN), and (4) hormonal receptor positive and HER-2 positive were defined as a triple positive group(TP).

### Statistical analysis

Descriptive statistics of the characteristics of the patients are presented as frequencies and percentages. The optimal cut-point for Ki-67 level was determined using receiver operating characteristic (ROC) curves according where the Youden’s index is maximum (using the *roctab* and *cutpt* command in Stata). The area under the curve (AUC), sensitivity, and specificity were also considered for the correctness of classification. The AUC values between 0.51 and 0.60, 0.61–0.70, 0.71–0.80, 0.81–0.90, and 0.90–1.00 were considered in terms of fail, poor, fair, good, and excellent discrimination ability, respectively [14].

The 8-year survival rate was estimated using the Kaplan-Meier method. The survival rate between patients with high and low Ki-67 level according to the provided cut-point was compared using the log-rank test. Cox regression analysis was performed to examine the associated between the Ki-67 level expression and 8-year survival in each of the breast cancer subtypes. A *p*-value of < 0.05 was considered statistically significant.

## Result

This retrospective study analyzed 507 invasive breast cancer patients with an age range of 21 to 90 years. The median follow-up time was 8 years (Interquartile Range, IQR:7.3-8), mean time follow up 6.9 years (SD:2.1). The patients’ characteristics are presented in Table 1. Out of the 507 breast cancer patients, 64% were aged between 41 and 60 years. Among them, 146 cases (28.8%) were diagnosed with stage 1 A, 129 cases (25.4%) with stage 2 A, and 24 cases (4.7%) with stage 3 C. Nodal negative disease was observed in 283 cases (56.8%), whereas 215 cases (43.2%) had nodal involvement of more than one node. The hormone- positive group constituted 35.5% of the patients, the HER-2 enriched group was 24.5%, the triple positive group was 27.4%, and the triple negative group was 12.6%.

**Table 1:** The characteristics of primary breast cancer patients diagnosed between January 2013 and December 2015 (*N* = 507)

	Frequencies (*N* = 507)	Percentages
Age		
< 41	66	13.0
41–60	328	64.7
> 60	113	22.3
Staging of breast cancer		
1A	146	28.8
1B	31	6.1
2A	129	25.4
2B	95	18.7
3A	53	10.5
3B	29	5.7
3C	24	4.7
LN metastasis / 9 (missing)		
Node negative	283	56.8
Node positive	215	43.2
Tumor Grade / 17 (missing)		
G1	15	3.1
G2	250	51.0
G3	225	45.9
Subtypes		
Hormone + (ER + PR + Her-2-)	180	35.5
HER-2 enrich (ER-PR-Her-2+)	124	24.5
Triple positive	139	27.4
Triple negative	64	12.6
Ki-67 level (%)		
0–14	129	25.4
15–30	158	31.2
31–45	59	11.6
> 45	161	31.8
Chemotherapy		
Antracylin	223	44.0
Taxane	154	30.4
No	130	25.6
HER2		
Used	52	10.3
No	455	89.7
Recurrent within 8 years after diagnosis breast cancer		
Yes	83	16.4
No	424	83.6
Dead within 8 years after diagnosis breast cancer		
Yes	97	19.1
No	410	80.9

**Fig. 1:**
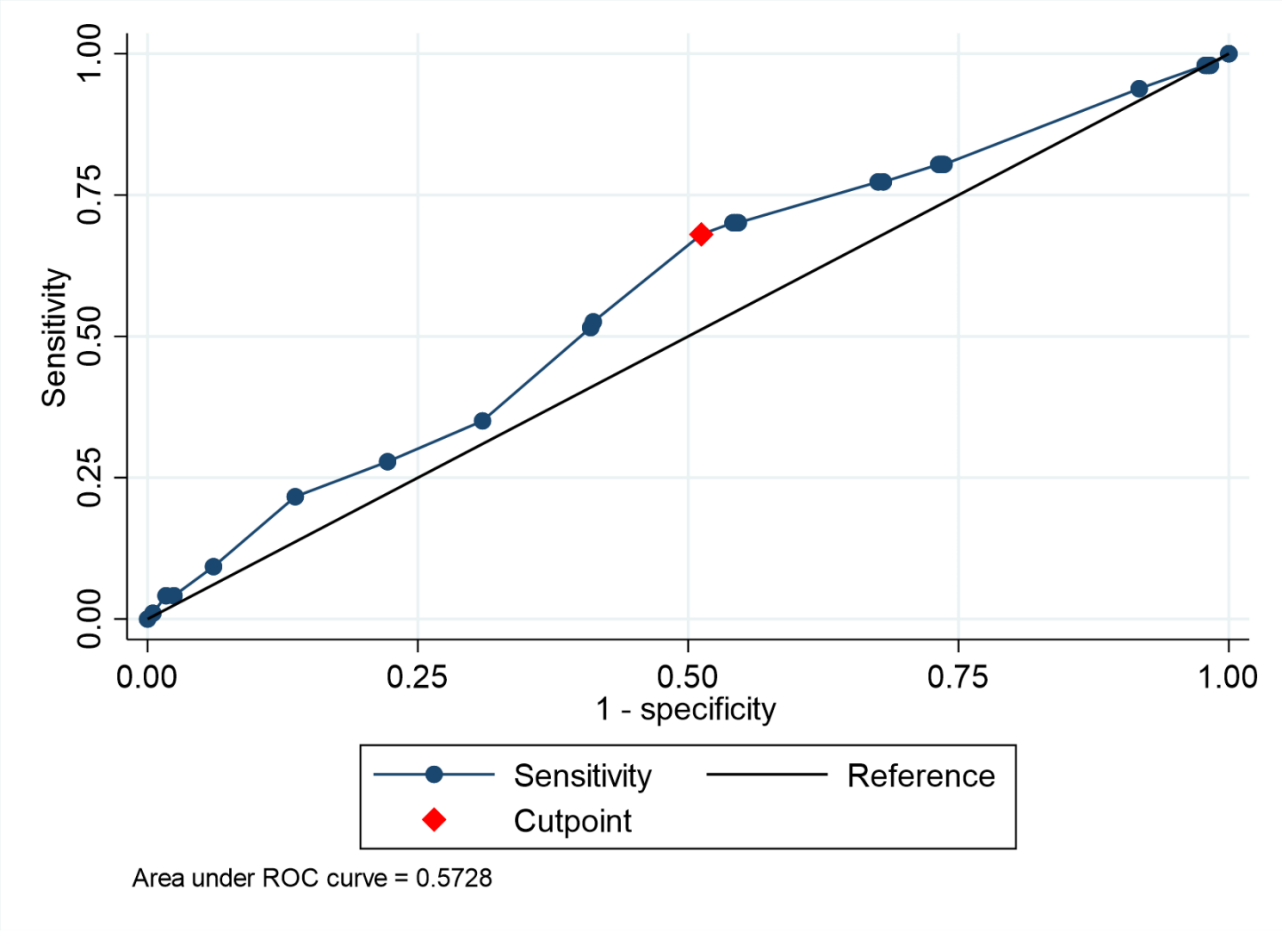
The ROC of the cut-point of Ki-67

**Fig. 2:**
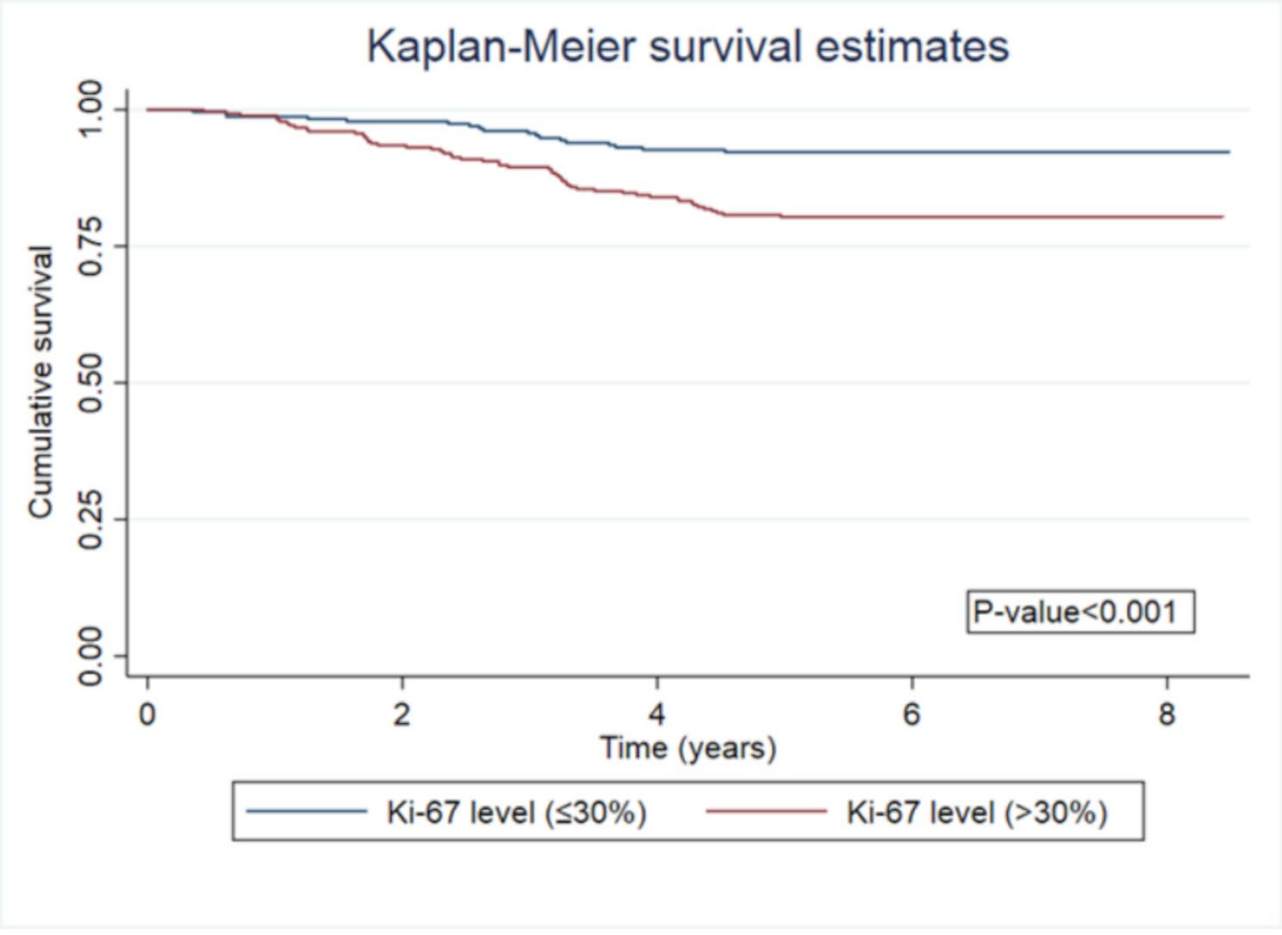
8-year survival of breast cancer patients

According to the Receiver Operating Characteristic (ROC) curve, the optimal cut-off point for Ki-67 was *≥* 30% for predicting mortality within 8 years, providing a sensitivity of 68% and a specificity of 48.78% with 52.47% correctness of classification. The area under the ROC curve was 0.5728 (Fig. 1).In Fig. 2, the survival analysis shown that the patient with a high Ki-67 level (*≥* 30%) had a significantly lower 8-year survival rate than a patient with low Ki-67 level (*p* < 0.001).

The univariable analysis in Table 2 show that age > 60 years (HR = 2.29, 95% *P* < 0.001), locally advanced staging (HR = 4.84, 95%, *P* < 0.001), nodal involvement at least 1 node (HR = 3.61, *P* < 0.001), Ki-67 *≥* 30 (HR = 1.94,,P 0.002) were associated with worse overall survival. After adjusting for other factors, age > 60 years (adjusted HR = 2.70, *P* < 0.001), locally advanced staging (adjusted HR = 3.37, *P* < 0.001), nodal involvement (adjusted HR = 2.97,*P* < 0.001), Ki-67 ≥ 30 (adjusted HR = 1.65, *P* = 0.04), and triple-negative subtype (adjusted HR = 2.01, 95% CI: 1.03–3.91, *P* = 0.04) remained significant predictors of worse overall survival. Meanwhile, chemotherapy with Taxane was associated with better overall survival (HR = 0.52, *P* = 0.01).

**Table 2:** Risk factor of the dead with breast cancer within 8 years

	Univariable			multivariable		
	HR	95%CI	*P*-value	aHR	95%CI	*P*-value
Age						
< 41	1.57	0.87–2.85	0.14	1.91	1.04–3.52	0.04
41–60	Reference	-	-	Reference	-	-
> 60	2.29	1.48–3.54	< 0.001	2.70	1.69–4.32	< 0.001
Staging						
Early stage (1 A-2B)	Reference	-	-	Reference	-	-
Locally advance (3 A-3 C)	4.84	3.25–7.21	< 0.001	3.37	2.02–5.61	< 0.001
Tumor Grade						
1	Reference	-	-	Reference	-	-
2	2.82	0.39–20.44	0.31	1.60	0.22–11.85	0.65
3	3.51	0.48–25.42	0.21	1.43	0.19–10.77	0.73
LN metastasis						
Node Negative	Reference	-	-	Reference	-	-
Node Positive	3.61	2.32–5.62	< 0.001	2.97	1.64–5.39	< 0.001
Ki67 (Cutoff median)						
< 30	Reference	-	-	Reference	-	-
≥ 30	1.94	1.26–2.97	0.002	1.65	1.03–2.66	0.04
Subtype						
Hormone positive	Reference	-	-	Reference	-	-
Her-2 enrich	1.54	0.93–2.55	0.09	1.53	0.88–2.65	0.13
Triple positive	0.80	0.45–1.41	0.43	0.59	0.32–1.10	0.10
Triple negative	1.72	0.95–3.11	0.07	2.01	1.03–3.91	0.04
Chemotherapy						
Receive anthracycline vs. Not receive	0.87	0.58–1.31	0.51	NA		
Receive Taxane vs. Not receive	1.33	0.88–2.01	0.18	0.52	0.32–0.86	0.01

This study conducted a thorough examination of the mortality risk linked to breast cancer, considering the stratification by cancer stage and subgroups defined by various factors. The results from the subgroup analysis revealed a significant finding in Table 3. Individuals aged over 60 faced a substantially higher risk of mortality within an 8-year period compared to their counterparts aged 41–60. This pattern was consistent for both early-stage and locally advanced breast cancer. The adjusted hazard ratios (aHR) were 1.97 (*P* = 0.03) for early-stage cases and 5.17 (*P* < 0.001) for locally advanced cases. Patients with positive lymph node metastasis exhibited a significantly higher risk of breast cancer-related death within 8 years compared to patients without lymph node involvement, as indicated by an aHR of 2.45 and a *p*-value of 0.002 in early-stage breast cancer. However, this effect was not observed in locally advanced-stage cases. No statistically significant difference was observed in the 8-year incidence of breast cancer mortality among subtypes in the early stage. However, in locally advanced stages, both the TN and HER2-enriched subtypes showed a higher susceptibility to breast cancer-related mortality compared to the hormone-positive group and TP subtype. Elevated Ki-67 expression was linked to an increased risk of breast cancer-related mortality in locally advanced breast cancer (HR 2.06, *P* = 0.04). In contrast, no statistically significant association was identified in the early stage.

**Table 3:** RiskRisk factor of the dead with breast cancer within 8 years divided by stage of breast cancer

	Univariable			multivariable		
	HR	95%CI	*P*-value	aHR	95%CI	*P*-value
Early stage (1 A-2B)						
Age						
< 41	1.16	0.48–2.82	0.74	1.11	0.46–2.70	0.81
41–60	Reference	-	-	Reference	-	-
> 60	1.81	0.97–3.35	0.06	1.97	1.06–3.67	0.03
LN metastasis						
Node Negative	Reference	-	-	Reference	-	-
Node Positive	2.40	1.37–4.20	0.002	2.45	1.40–4.29	0.002
Tumor Grade						
1	Reference	-	-	NA		
2	1.59	0.22–11.73	0.65			
3	1.65	0.22–12.25	0.63			
Subtype						
Hormone positive	Reference	-	-	NA		
Her-2 enrich	1.05	0.51–2.14	0.90			
Triple positive	0.75	0.36–1.57	0.44			
Triple negative	0.92	0.37–2.28	0.85			
Ki67 (Cut of median)						
< 30	Reference	-	-	Reference	-	-
≥ 30	1.43	0.81–2.52	0.22	1.49	0.84–2.63	0.17
Locally advance (3 A-3 C)						
Age						
< 41	2.38	1.05–5.37	0.04	4.55	1.85–11.17	0.001
41–60	Reference	-	-	Reference	-	-
> 60	3.38	1.81–6.31	< 0.001	5.17	2.59–10.35	< 0.001
LN metastasis						
Node Negative	Reference	-	-			
Node Positive	0.89	0.28–2.88	0.85	NA		
Subtype						
Hormone positive	Reference	-	-	Reference	-	-
Her-2 enrich	1.72	0.81–3.65	0.16	2.12	0.96–4.71	0.06
Triple positive	0.73	0.29–1.82	0.50	0.39	0.15–1.02	0.06
Triple negative	3.76	1.62–8.72	0.002	5.21	2.02–13.44	0.001
Ki67 (Cut of median)						
< 30	Reference	-	-	Reference	-	-
≥ 30	2.06	1.05–4.05	0.04	3.09	1.45–6.61	0.004

**Fig. 3:**
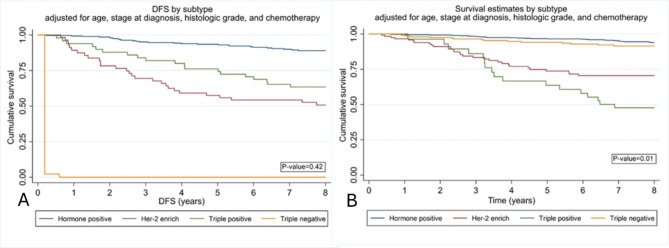
Kaplan-Meier graph estimate DFS and OS according by tumor subtype. **A**: DFS disease free survival **B**: 8-year survival

The Overall Survival (OS) and Disease-Free Survival (DFS) were illustrated using the Kaplan-Meier curve, with a median follow-up duration of 8 years. The findings indicate that the group characterized by hormone positivity exhibited the most favorable DFS, contrasting with the Triple-Negative (TN) group, which displayed the least favorable DFS. However, this distinction did not achieve statistical significance (*p*-value = 0.42). Conversely, the hormone-positive cohort demonstrated the highest OS, while the Triple-Positive (TP) group recorded the lowest OS, and this difference reached statistical significance (*p*-value = 0.01). (Fig. 3 )

**Fig. 4:**
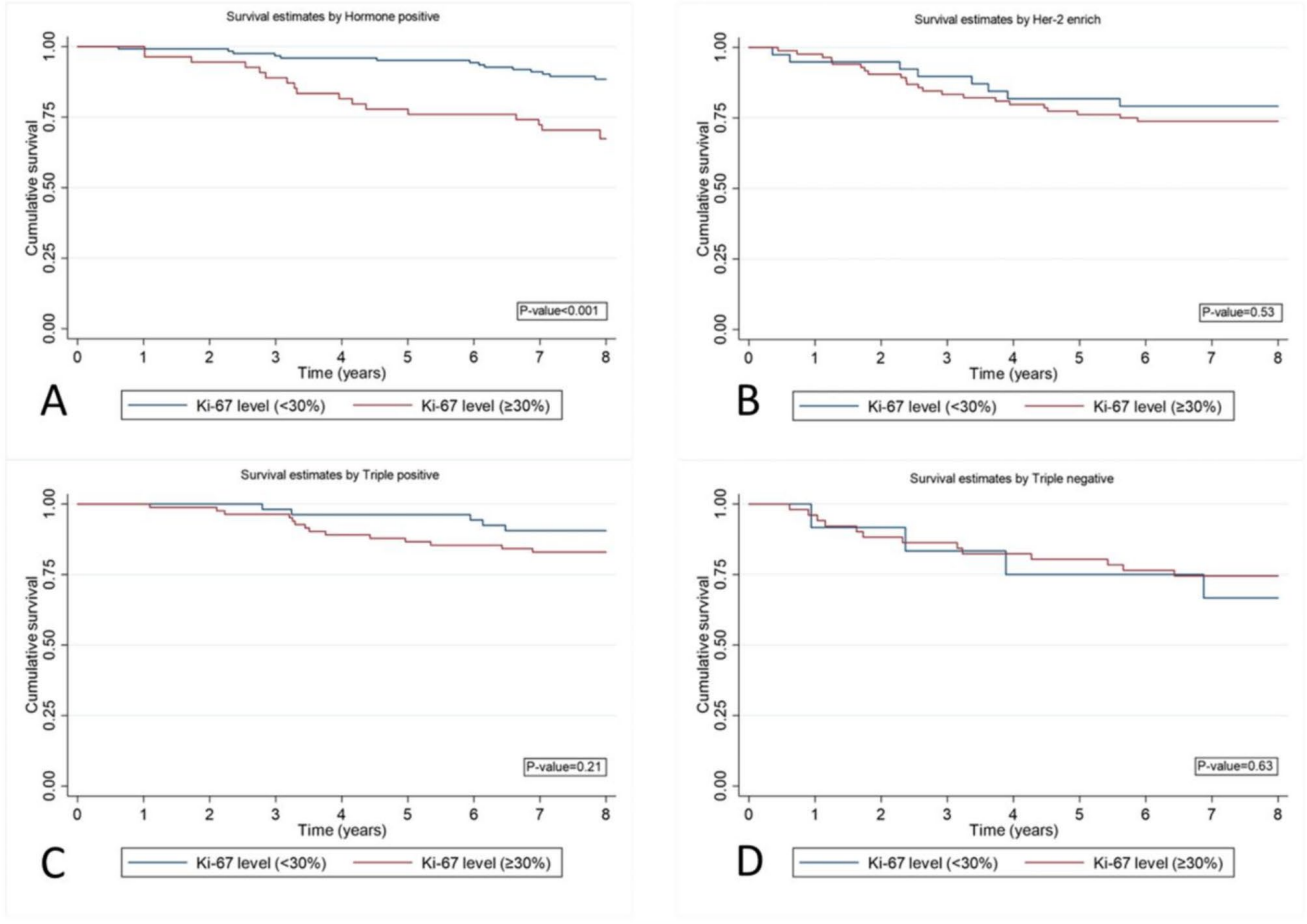
Kaplan-Meier curves estimate OS estimate by subtype and Ki-67 level. **A**: hormone positive, **B**: Her2 enrich **C**: triple positive **D**: triple negative

In the investigation of KI-67 levels associated with each breast cancer subtype, it was determined that elevated Ki-67 level (≥ 30%) had a significantly lower 8-year survival rate in HR + group (*p* < 0.001). However, KI-67 levels did not have a statistically significantly different OS in the HER2, TP and TN groups (Fig. 4).

**Fig. 5:**
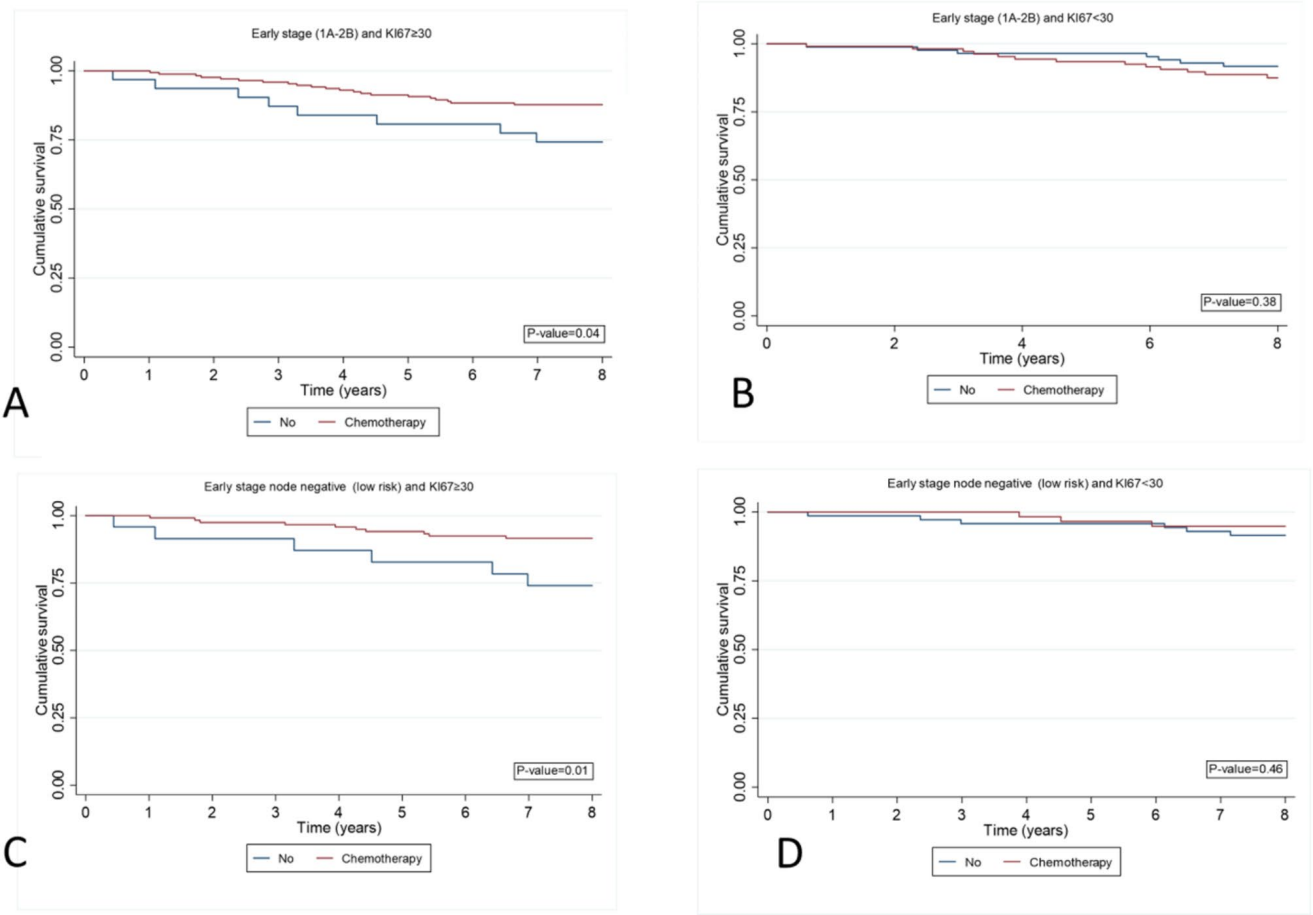
Kaplan-Meier curves estimate overall survival (OS) according received or not received chemotherapy in early stage breast cancer. Early stage breast cancer **A**: Ki-67 ≥ 30 and **B**: Ki-67 < 30. Early stage breast cancer without nodal involvement **C**: Ki-67 ≥ 30 and **D**: Ki-67 < 30

In the subgroup analysis concerning the association between Ki-67 expression and chemotherapy in early-stage patients, it was found that patients who received chemotherapy exhibited significantly higher survival compared to those who did not. This effect was notable only in patients with high Ki-67 (≥ 30). However, in patients with low Ki-67, chemotherapy did not impact overall survival. Upon further analysis of early-stage patients with negative lymph nodes, a significant survival advantage was observed for patients with high Ki-67 expression (≥ 30) who received chemotherapy compared to those who did not. Conversely, a similar effect of chemotherapy on overall survival was not observed in patients with low Ki-67 expression within this nodal-negative early-stage cancer subgroup (Fig. 5).

**Fig. 6:**
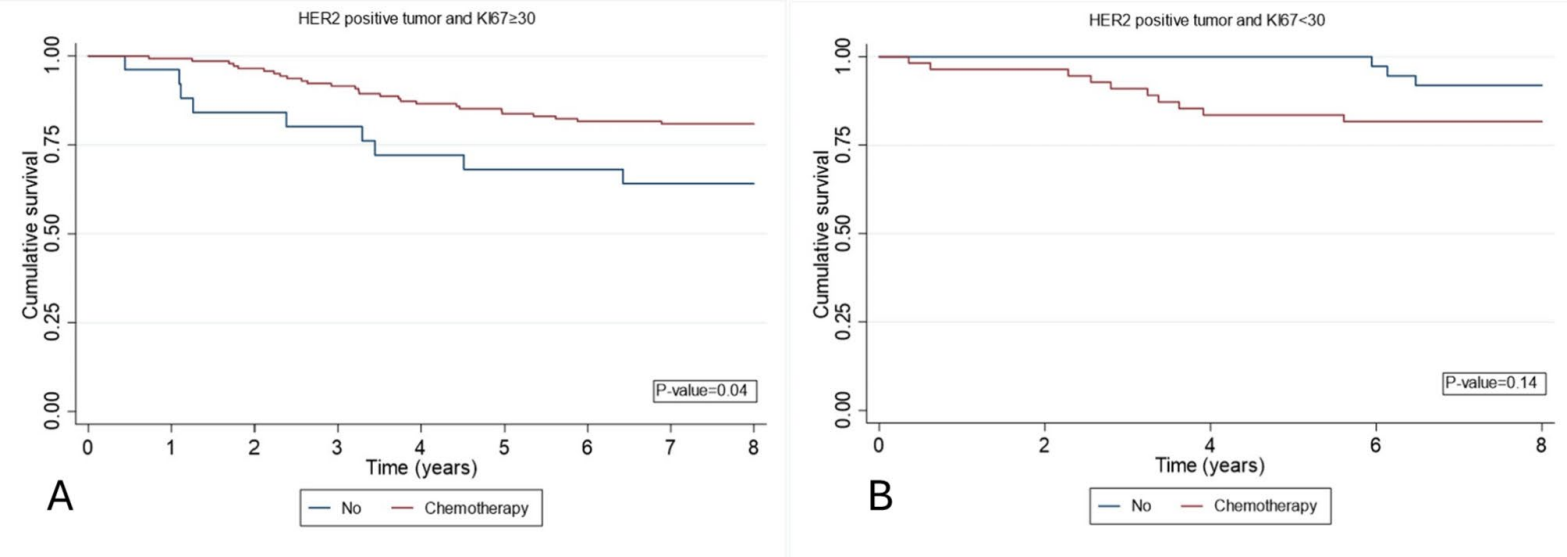
Kaplan-Meier curves estimate OS according received or not received chemotherapy in HER-2 positive breast cancer. **A**: Ki-67 ≥ 30, **B**: Ki-67 < 30

**Fig. 7:**
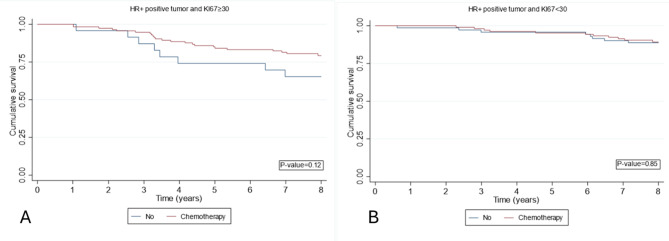
Kaplan-Meier curves estimate OS according received or not received chemotherapy in hormone positive breast cancer. **A**: Ki-67 ≥ 30, **B**: Ki-67 < 30

In the subgroup analysis of HER2-positive patients (including TP subtype and HER2-enriched subtype), a notable survival advantage was observed in patients with high KI-67 expression who received chemotherapy compared to those who did not (Fig. 6). Similarly, in the subgroup analysis of HR + patients, a similar effect was noted, although this difference did not reach statistical significance( Fig. 7).

## Discussion

This investigation revealed the pivotal role of Ki-67 expression as a prognostic determinant for the 8-year survival of individuals with breast cancer. This discovery aligns with findings from several previous studies [2, 4, 15].Furthermore, our study specified the optimal cut-point level of Ki-67 for patients in northern Thailand, determined to be 30%. This determination is consistent with the results of an inquiry by Yue Hu et al. [10], examining the optimal Ki-67 cut-point for Chinese breast cancer patients. It is essential to note, however, that various prior studies have proposed an alternative optimal cut-point of 20% [7, 8] The discrepancy in cut-off values between our study and others likely arises from the differing endpoints used to determine prognostic significance; our study employed overall survival, whereas others focused on disease-specific survival. Additionally, the St. Gallen Consensus in 2011 established a Ki-67 cut-point of 14% for categorizing luminal A subtypes, using gene expression testing as the gold standard. This cut-point was intended for classification purposes, not for determining prognostic value. By 2015, the St. Gallen Consensus reflecting variability in testing methods and patient populations and recommended using median Ki-67 value from local laboratories as the preferred cut-point.

In our investigation into the prognostic implications of Ki-67 levels, we made a significant observation regarding overall survival. Our findings suggest that breast cancer patients with a higher Ki-67 level (*≥* 30%) experienced more adverse survival outcomes compared to those with lower Ki-67 expression. These observations align with previous studies in the field, strengthening the proposition that elevated Ki-67 expression is associated with an inferior prognosis among breast cancer patients [5, 16]. 

In our subgroup analysis stratified by cancer stage, we identified that high Ki-67 level is a significant risk factor of dead within 8 years, particularly in cases of locally advanced cancer. However, intriguingly, this effect did not achieve statistical significance in the context of early-stage cancer. These observations contrast with findings in other studies [17, 18] emphasizing the substantial impact of Ki-67 in early-stage or low-aggressive cancer. It is plausible that the divergence in results between our study and prior investigations may be attributed to the limited sample size in our research. Furthermore, discrepancies in defining low and high Ki-67 expression levels across studies could lead to variations. In previous studies, low Ki-67 was defined as 5% or less, and high Ki-67 as 30% or more. In our study, however, we operationalized low Ki-67 expression as ranging from 0 to 29%. This difference in defining cutoff points for Ki-67 levels might be a potential factor contributing to the observed variation in effects.

Previous research has explored the association between Ki-67 and other low-aggressive prognostic factors. For example, a study by Qin Liang et al. [18] demonstrated a relationship between low histologic grade and Ki-67 expression. Similarly, Fallah P et al. [3] found that among patients with low Oncotype DX scores, those with high Ki-67 levels had a significantly higher relapse rate compared to those with low Ki-67 levels. Our findings align with these studies. In the subgroup analysis of low-aggressive tumors, specifically early-stage breast cancer with negative lymph nodes, patients with high Ki-67 levels showed significantly lower disease-free survival (DFS) compared to those with low Ki-67 levels. Moreover, among patients with early-stage breast cancer, negative lymph nodes, and high Ki-67 levels, chemotherapy was associated with a reduced recurrence rate compared to patients who did not receive chemotherapy. These results suggest that Ki-67 expression may serve as an important prognostic factor in low-aggressive tumors. Our study underscores the value of considering Ki-67 levels when making treatment decisions for very low-risk patients. For these patients, additional evaluation with tools such as Oncotype DX or MammaPrint may be beneficial in refining treatment strategies. However, in settings where access to advanced diagnostic tools is limited, this study supports using Ki-67 as a practical marker to guide chemotherapy decisions.

In tumors characterized by a more aggressive phenotype, the impact of Ki-67 may show a reduced prominence and lack statistical significance. This result can be attributed to the heightened aggressiveness associated with clinical features such as nodal positivity, larger tumor size, higher tumor grade, and a high Oncotype Dx score. Consequently, the expression level of Ki-67 may not exert a substantial effect in the context of these aggressive tumor characteristics.

In our study, we observed that age above 60 years, lymph node involvement, and locally advanced disease and high Ki-67 were identified as predictors of poorer prognostic outcomes for breast cancer patients. Additionally, we found that triple-negative breast cancer exhibited a poorer prognosis compared to the triple-positive or hormone-positive groups. These findings are consistent with previous studies conducted in the field [16, 19–21].

There are some limitations in this study. Firstly, the AIC of the ROC curve according to the Ki-67 classification was noticeably low. Further study to examine the ability of classification in other settings or using other indices should be conducted. Second, since the correctness of classification of the purposed cut-off point was 52.47% (sensitivity of 68.04% and specificity of 48.78%), using this cut-off point only should be caution. The mortality risk should be also considered for other potential associated factors. Third, the cut-off point in this study was specific to the Northern Thailand region and may not be applicable to populations with different genetic backgrounds or healthcare systems. External validation of this cut-off point should be conducted.

## Conclusion

The optimal cut point for the KI-67 level in Northern Thailand is identified as 30%. Patients with a KI-67 level exceeding 30% demonstrate a significantly lower 8-year survival rate compared to those with a KI-67 level below 30%. In low-risk patients, particularly those with hormonal subtypes or in the early stage with nodal negativity, the Ki-67 level emerges as a crucial factor to be considered in determining appropriate treatment for breast cancer patients. Our study is in alignment with other research, emphasizing the importance of the Ki-67 index as a prognostic factor in the planning of suitable treatment for low-risk breast cancer patients.

## Data Availability

The datasets generated for this current study are available from the corresponding author on reasonable request.

## Abbreviations

AXL: Axillary lymph node
Ki-67 LI: Ki-67 level index
ER: Estrogen receptor
PR: Progesterone receptor
LSAB: Labeled streptavidin-biotin
ASCO: American Society of Clinical Oncology
ROC: Receiver operating characteristic
AUC: Area under the curve
OS: Overall survival
DFS: Disease- free survival
TN: Triple -Negative
TP: Triple positive

## References

[1] Sung H, Ferlay J, Siegel RL, Laversanne M, Soerjomataram I, Jemal A, et al. Global Cancer statistics 2020: GLOBOCAN estimates of incidence and Mortality Worldwide for 36 cancers in 185 countries. CA Cancer J Clin. 2021;71(3):209–49. 33538338 10.3322/caac.21660

[2] Nishimura R, Osako T, Okumura Y, Hayashi M, Toyozumi Y, Arima N. Ki-67 as a prognostic marker according to breast cancer subtype and a predictor of recurrence time in primary breast cancer. Exp Ther Med. 2010;1(5):747–54. 22993598 10.3892/etm.2010.133 PMC3445951

[3] Fallah P, Mulla NK, Aloyz R, Aleynikova O, Florea A, Pelmus M, et al. Can high Ki67 predict distant recurrence in early-stage breast cancer with low Oncotype Dx score? J Clin Oncol. 2021;39(15suppl):e12561–e.

[4] de Azambuja E, Cardoso F, de Castro G Jr., Colozza M, Mano MS, Durbecq V, et al. Ki-67 as prognostic marker in early breast cancer: a meta-analysis of published studies involving 12,155 patients. Br J Cancer. 2007;96(10):1504–13. 17453008 10.1038/sj.bjc.6603756 PMC2359936

[5] Stuart-Harris R, Caldas C, Pinder SE, Pharoah P. Proliferation markers and survival in early breast cancer: a systematic review and meta-analysis of 85 studies in 32,825 patients. Breast. 2008;17(4):323–34. 18455396 10.1016/j.breast.2008.02.002

[6] Goldhirsch A, Wood WC, Coates AS, Gelber RD, Thürlimann B, Senn HJ. Strategies for subtypes–dealing with the diversity of breast cancer: highlights of the St. Gallen International Expert Consensus on the primary therapy of early breast Cancer 2011. Ann Oncol. 2011;22(8):1736–47. 21709140 10.1093/annonc/mdr304 PMC3144634

[7] Cheang MC, Chia SK, Voduc D, Gao D, Leung S, Snider J, et al. Ki67 index, HER2 status, and prognosis of patients with luminal B breast cancer. J Natl Cancer Inst. 2009;101(10):736–50. 19436038 10.1093/jnci/djp082 PMC2684553

[8] Bustreo S, Osella-Abate S, Cassoni P, Donadio M, Airoldi M, Pedani F, et al. Optimal Ki67 cut-off for luminal breast cancer prognostic evaluation: a large case series study with a long-term follow-up. Breast Cancer Res Treat. 2016;157(2):363–71. 27155668 10.1007/s10549-016-3817-9 PMC4875067

[9] Tashima R, Nishimura R, Osako T, Nishiyama Y, Okumura Y, Nakano M, et al. Evaluation of an optimal cut-off point for the Ki-67 index as a prognostic factor in primary breast Cancer: a retrospective study. PLoS ONE. 2015;10(7):e0119565–e. 26177501 10.1371/journal.pone.0119565 PMC4503758

[10] Lombardi A, Lazzeroni R, Bersigotti L, Vitale V, Amanti C. The proper Ki-67 cut-off in hormone responsive breast Cancer: a Monoinstitutional Analysis with Long-Term Follow-Up. Breast Cancer (Dove Med Press). 2021;13:213–7. 33854368 10.2147/BCTT.S305440 PMC8039013

[11] Hu Y, Gu R, Zhao J, Yang Y, Liu F, Jin L, et al. Prognostic significance of Ki67 in Chinese women diagnosed with ER+/HER2– breast cancers by the 2015 St. Gallen consensus classification. BMC Cancer. 2017;17(1):28. 28061893 10.1186/s12885-016-3021-7 PMC5219721

[12] Coates AS, Winer EP, Goldhirsch A, Gelber RD, Gnant M, Piccart-Gebhart M, et al. Tailoring therapies–improving the management of early breast cancer: St Gallen International Expert Consensus on the primary therapy of early breast Cancer 2015. Ann Oncol. 2015;26(8):1533–46. 25939896 10.1093/annonc/mdv221 PMC4511219

[13] Goldhirsch A, Winer EP, Coates AS, Gelber RD, Piccart-Gebhart M, Thürlimann B, et al. Personalizing the treatment of women with early breast cancer: highlights of the St Gallen International Expert Consensus on the primary therapy of early breast Cancer 2013. Ann Oncol. 2013;24(9):2206–23. 23917950 10.1093/annonc/mdt303 PMC3755334

[14] Li F, He H. Assessing the Accuracy of Diagnostic tests. Shanghai Arch Psychiatry. 2018;30(3):207–12. 30858674 10.11919/j.issn.1002-0829.218052 PMC6410404

[15] Soliman NA, Yussif SM. Ki-67 as a prognostic marker according to breast cancer molecular subtype. Cancer Biol Med. 2016;13(4):496–504. 28154782 10.20892/j.issn.2095-3941.2016.0066 PMC5250608

[16] Zhu X, Chen L, Huang B, Wang Y, Ji L, Wu J, et al. The prognostic and predictive potential of Ki-67 in triple-negative breast cancer. Sci Rep. 2020;10(1):225. 31937819 10.1038/s41598-019-57094-3 PMC6959292

[17] Yerushalmi R, Woods R, Ravdin PM, Hayes MM, Gelmon KA. Ki67 in breast cancer: prognostic and predictive potential. Lancet Oncol. 2010;11(2):174–83. 20152769 10.1016/S1470-2045(09)70262-1

[18] Dowsett M, Nielsen TO, A’Hern R, Bartlett J, Coombes RC, Cuzick J, et al. Assessment of Ki67 in breast cancer: recommendations from the International Ki67 in breast Cancer working group. J Natl Cancer Inst. 2011;103(22):1656–64. 21960707 10.1093/jnci/djr393 PMC3216967

[19] Nielsen TO, Leung SCY, Rimm DL, Dodson A, Acs B, Badve S, et al. Assessment of Ki67 in breast Cancer: updated recommendations from the International Ki67 in breast Cancer Working Group. J Natl Cancer Inst. 2021;113(7):808–19. 33369635 10.1093/jnci/djaa201 PMC8487652

[20] Pathmanathan N, Balleine RL, Jayasinghe UW, Bilinski KL, Provan PJ, Byth K, et al. The prognostic value of Ki67 in systemically untreated patients with node-negative breast cancer. J Clin Pathol. 2014;67(3):222–8. 24403187 10.1136/jclinpath-2013-201793

[21] Łukasiewicz S, Czeczelewski M, Forma A, Baj J, Sitarz R, Stanisławek A. Breast Cancer-epidemiology, risk factors, classification, prognostic markers, and current treatment Strategies-An updated review. Cancers (Basel). 2021;13(17). 10.3390/cancers13174287 PMC8428369 34503097

